# ﻿*Paragoniastreavariabilis* Kishi, Nomura & Fukami, sp. nov. (Cnidaria, Anthozoa, Scleractinia), a new coral species previously considered as a variant of *Paragoniastreadeformis*, from Japan and northern Taiwan

**DOI:** 10.3897/zookeys.1205.121507

**Published:** 2024-06-24

**Authors:** Daigo Kishi, Keiichi Nomura, Yoko Nozawa, Seiji Arakaki, Hironobu Fukami

**Affiliations:** 1 Graduate School of Agriculture, University of Miyazaki, Miyazaki 889-2155, Japan University of Miyazaki Miyazaki Japan; 2 Sabiura Marine Park Research Station, Kushimoto, Wakayama 649-3514, Japan Sabiura Marine Park Research Station Wakayama Japan; 3 Biodiversity Research Center, Academia Sinica, Taipei 115, Taiwan Biodiversity Research Center, Academia Sinica Taipei Taiwan; 4 Tropical Biosphere Research Center, University of the Ryukyus, Okinawa 905-0227, Japan University of the Ryukyus Okinawa Japan; 5 Department of Marine Science, Faculty of Fisheries and Marine Science, Universitas Diponegoro, Semarang, Indonesia Universitas Diponegoro Semarang Indonesia; 6 Amakusa Marine Biological Laboratory, Kyushu University, Kumamoto 863-2507, Japan Kyushu University Kumamoto Japan; 7 Department of Marine Biology and Environmental Sciences, Faculty of Agriculture, Miyazaki University, Miyazaki 889-2155, Japan Miyazaki University Miyazaki Japan

**Keywords:** Merulinidae, molecular phylogeny, taxonomy, temperate region, zooxanthellate scleractinian corals

## Abstract

A new zooxanthellate scleractinian coral, *Paragoniastreavariabilis* Kishi, Nomura & Fukami, **sp. nov.** (Scleractinia, Merulinidae), is described from non-coral reef regions of Japan and northern Taiwan. This new species was previously recognized as a morphological variant of *Paragoniastreadeformis* (Veron, 1990) and can be morphologically distinguished from that species by lacking groove-and-tube structures on corallite wall joints, and by having larger calices, numerous septa, and up to three corallites in one valley. The new species also formed an independent clade from its congeners, *P.australensis* (Milne Edwards & Haime, 1857), *P.deformis* and *P.russelli* (Wells, 1954), in the molecular phylogeny based on the mitochondrial intergenic region and nuclear ribosomal internal transcribed spacers.

## ﻿Introduction

The zooxanthellate scleractinian coral genus *Paragoniastrea* Huang, Benzoni & Budd, 2014, which belongs to the family Merulinidae Verrill, 1865, inhabits the Indo-Pacific region (Huang et al. 2014). Currently, this genus contains three species, namely, *P.australensis* (Milne Edwards & Haime, 1857), *P.deformis* (Veron, 1990), and *P.russelli* (Wells, 1954), which were transferred from other merulinid genera, *Favites* or *Goniastrea*, to this genus based on molecular phylogenetic and morphological data (Huang et al. 2014). *Paragoniastrea* is characterized by encrusting or massive colony morphology, plocoid, cerioid or meandroid in corallite arrangements, and developed paliform lobes. These characteristics are similar to those of *Favites* and *Goniastrea*, but *Paragoniastrea* is distinguished from these genera by its distinct phylogenetic affinities (Huang et al. 2014; Quek et al. 2023) and several morphological differences in corallite characteristics, such as the height of calice relief, septa (number, tooth height, and spacing), and theca structure (Huang et al. 2014).

*Paragoniastreadeformis*, described from Kushimoto, Wakayama, mainland Japan, is mainly distributed around the warm-temperate, non-coral reef region of Japan (Veron 1990, Nishihira and Veron 1995). Although *P.deformis* has also been reported from the Philippines (Wilfredo and Emmi 2003), whether it is the same species or not remains unknown because the specimen (P1L01316) shown by Wilfredo and Emmi (2003) has corallites approximately twice as large as the holotype and has up to three distinct corallites in a valley between corallite walls. *Paragoniastreadeformis* is known to have groove-and-tube structures (groove-and-tubercle structure *sensu*Rosen 1968) on corallite wall joints. Groove-and-tube structure is defined as a feature of vertical and horizontal intercommunicating holes between adjacent corallites, some of which are formed around corallites by parasitic worms (Rosen 1968; Randall and Eldredge 1976). This character has been used as a specific morphological characteristic in some species, including *P.deformis* and *Favitesvalenciennesii* (Milne Edwards & Haime, 1849) (Veron 1990). However, some colonies of *P.deformis* do not have any groove-and-tube structures, and they are considered to be intraspecific morphological variations of *P.deformis* (Nishihira 1991; Uchida and Soyama 1994; Nishihira and Veron 1995; Veron 2000; Veron et al. 2016). Huang et al. (2014) reported that two specimens of *P.deformis* (*Paragoniastrea* sp.) with a non-groove-and-tube type were separated from typical *P.deformis* with groove-and-tube structures by molecular phylogenetic analysis. To date, morphological and molecular phylogenetic analyses for the non-groove-and-tube type of *P.deformis* remain insufficient to define it as a species different from typical *P.deformis*. Groove-and-tube structure is recognized as one of the key species-specific morphological characters in *Favitesvalenciennesii*. Thus, this structure might be a good key character to define species in this genus.

This study has shown that the non-groove-and-tube type of *P.deformis* is distinct from *P.deformis* in both morphology and phylogeny, and describes a new species, *Paragoniastreavariabilis* sp. nov. In addition, three specimens without groove-and-tube structures but distinguished from *P.variabilis* sp. nov. by their distinct phylogenetic affinities and slight morphological differences were tentatively designated as P.aff.deformis as it is not yet clear if they represent a different species or not from *P.deformis*.

## ﻿Materials and methods

### ﻿Materials

**Figure 1.**
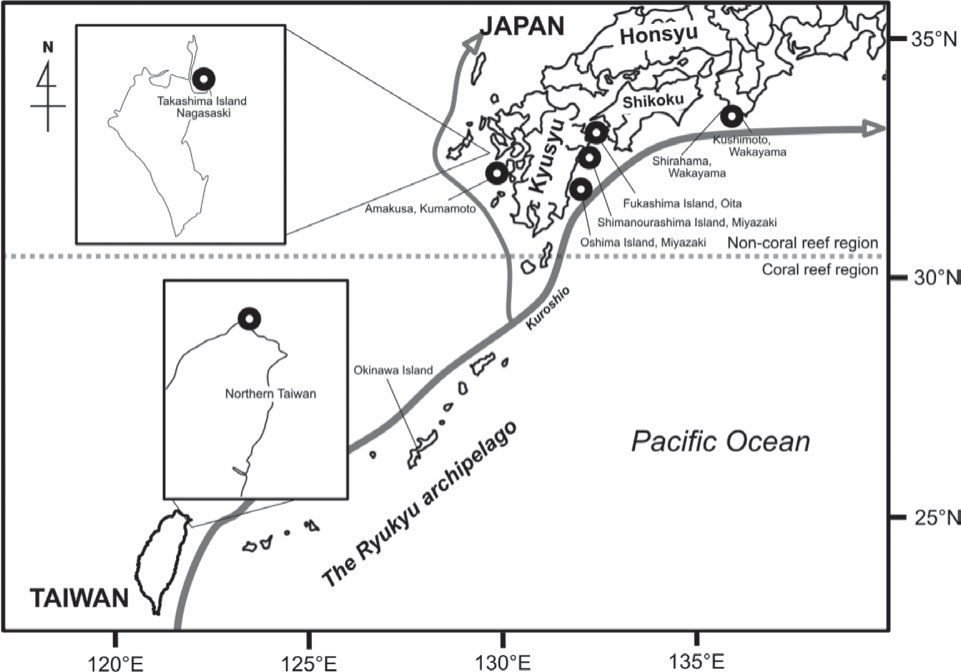
Sampling sites (black circles) where we collected skeletal and genetic samples in this study.

Sampling of materials was conducted at seven sites (Shirahama and Kushimoto, Wakayama; Fukashima Island, Oita; Takashima Island, Nagasaki; Shimanourashima Island and Oshima Island, Miyazaki; Amakusa, Kumamoto) in Japan and at one location (Yehliu, New Taipei) in northern Taiwan (Fig. 1, Suppl. material 1). For comparison with the new species, we examined the holotype of *P.deformis* (MTQ G61876) and the photographic images of holotypes of *P.australensis* (MNHN IK-2010-409) and *P.russelli* (USNM 45004). Specimens of other *Paragoniastrea* species collected at the same sites were also used (Suppl. material 1). Samples were collected by a hammer and chisel to break only the necessary size (around 10 cm^3^) for morphological analysis.

A small piece (<1 cm^3^) was also cut from each collected sample with nippers for molecular phylogenetic analysis and was mixed with a guanidine solution (4M guanidine thiocyanate, 0.1% sodium N-laurylsarcosine, 10 mM Tris-HCl pH 8.0, 0.1M 2-mercaptoethanol; Fukami et al. 2004) to dissolve tissues. The remaining samples were bleached and labeled with numbers associated with the collection sites, and they were stored as skeletal specimens for use in morphological analysis.

The holotype and two paratypes of *Paragoniastreavariabilis* sp. nov. have been deposited in the Department of Marine Biology and Environmental Sciences, University of Miyazaki (MUFS) in Miyazaki, Japan. The other specimens were deposited in the following institutions: Seto Marine Biological Laboratory (SMBL), Wakayama, Japan; Sabiura Marine Park Research Station (SMP), Wakayama, Japan, and Zoological Collection, Biodiversity Research Museum, Academia Sinica (ASIZC), Taipei, Taiwan. Museum abbreviations for type materials are as follows: Muséum national d’Histoire naturelle de Paris (MNHN, France), Museum of Tropical Queensland (MTQ, Australia), and National Museum of Natural History, Smithsonian Institution (USNM, USA).

### ﻿Morphological analysis

Measurements and observations of the skeletal specimens from Japan were made using a digital microscope (Keyence VHS-1000; WRAYMER WRAYCAM-NOA630 + Leica MZ16) and a scanning electron microscope (Hitachi High-Tech TM-1000). Taiwan specimens were photographed by a digital camera (OM system Tough TG-6) and the skeletal image was measured by Image J (version 1.5.3, https://imagej.net/ij/index.html).

To analyze the morphological differences between *P.variabilis* sp. nov. and *P.deformis*, morphological characteristics were compared using the Brunner-Munzel test or the Welch t-test, after confirming normality and homogeneity. In addition, principal component analysis (PCA) was performed on morphological data of *P.variabilis* sp. nov., *P.deformis* and P.aff.deformis, which were measured and standardized in this study. PCA was performed using the prcomp function in R version 4.3.1 (The R Foundation for Statistical Computing http://www.R-project.org). The PCA result was plotted using the R packages *devtools* and *ggbiplot*. The specimens shown in Suppl. material 1 were used in these analyses.

### ﻿Molecular phylogenetic analysis

Total genomic DNA was extracted from each guanidine sample by the conventional phenol-chloroform extraction method. Using the extracted DNA, mitochondrial intergenic region (IGR) and nuclear ribosomal internal transcribed spacers (ITS) were amplified by the PCR method. Primers used for each marker were MCOIF3 (5’- CCA AGA CGA TAT TTC GGA CTT -3’) and tRNAmetR (5’- GTG AGA CTC GAA CTC ACT TTT TT -3’) for IGR (Mitsuki et al. 2021), 1S (5’- GGG TAC CCT TTG TAC ACA CAC CGC CCG TCG CT -3’) and 2SS (5’- GCT TTG G GC GGC AGT CCC AAG CAA CCC GAC TC -3’) for ITS (Wei et al. 2006). PCR was performed in each sample using 11.32 µL of sterile distilled water, 2 µL of 10×buffer, 2 µL of forward primer, 2 µL of reverse primer, 1.6 µL of dNTPs, 0.08 µL of exTaq and 1 µL of DNA of each sample. PCR conditions for ITS and IGR markers were 94 °C for 60 s followed by 35 cycles at 94 °C for 30 s, 55 °C for 45 s, and 72 °C for 90 s, and 72 °C for 5 mins for the final extension. However, for some samples with unsuccessful amplification, we used the following PCR conditions: 94 °C for 60 s, 94 °C for 30 s, 48 °C for 45 s, and 72 °C for 90 s, repeated 5 times, followed by 94 °C for 30 s, 55 °C for 45 s, and 72 °C for 90 s, repeated 30 times, and 72 °C for 5 mins for the final extension. The amplified PCR products were treated with shrimp alkaline phosphatase and exonuclease I at 37 °C for 40 mins and 80 °C for 20 mins.

DNA sequences were determined by direct sequencing using ABI3730 sequencers by a contracted research service (FASMAC Co. Ltd., Kanagawa, Japan). Alignments of IGR and ITS sequences were carried out using the E-INS-i option in MAFFT 7 online (https://mafft.cbrc.jp/alignment/server/) (Kuraku et al. 2013; Katoh et al. 2019) under default parameters. Molecular phylogenetic trees were reconstructed using the neighbor-joining (NJ) and maximum-likelihood (ML) methods after all indels were deleted in MEGA ver. 11.0 (Tamura et al. 2021). MEGA was also used to estimate a model of nucleotide evolution for each marker (HKY+G for IGR, T92+G for ITS markers) and to conduct a bootstrap analysis (with 1000 replicates). All DNA sequences obtained in this study were submitted to the DNA Data Bank of Japan (DDBJ) (accession numbers LC804981–LC804999 for IGR, LC801365–LC801368, LC801370–LC801379 for ITS; Suppl. material 1).

The published DNA sequences of *Paragoniastrea* species used by Huang et al. (2014) for IGR and ITS were used to compare with our data (Suppl. material 2). There were many multi-peaks in the DNA sequences of ITS for two samples (sample numbers: JP009, JP065) used by Huang et al. (2014). Therefore, we newly extracted genomic DNA from one guanidine sample (JP065), which is stored at the University of Miyazaki and used in the analysis for ITS. For both the IGR and ITS trees, DNA sequences (accession numbers KJ666508 and KJ666509 for IGR, KJ666394 and KJ666396 for ITS) of *Merulinaampliata* from Huang et al. (2014) were used as outgroups.

## ﻿Taxonomy account

**Family Merulinidae Milne Edwards & Haime, 1857**

**Genus *Paragoniastrea* Huang, Benzoni & Budd, 2014**

### ﻿ Paragoniastrea variabilis

Taxon classificationAnimaliaScleractiniaMerulinidae

Kishi, Nomura & Fukami sp. nov.

4C677459-F70A-545F-95A8-EE61EB9BE76F

https://zoobank.org/CD11CAA7-81EA-4241-8DAE-BB1F80F5E406

**Figure 2.**
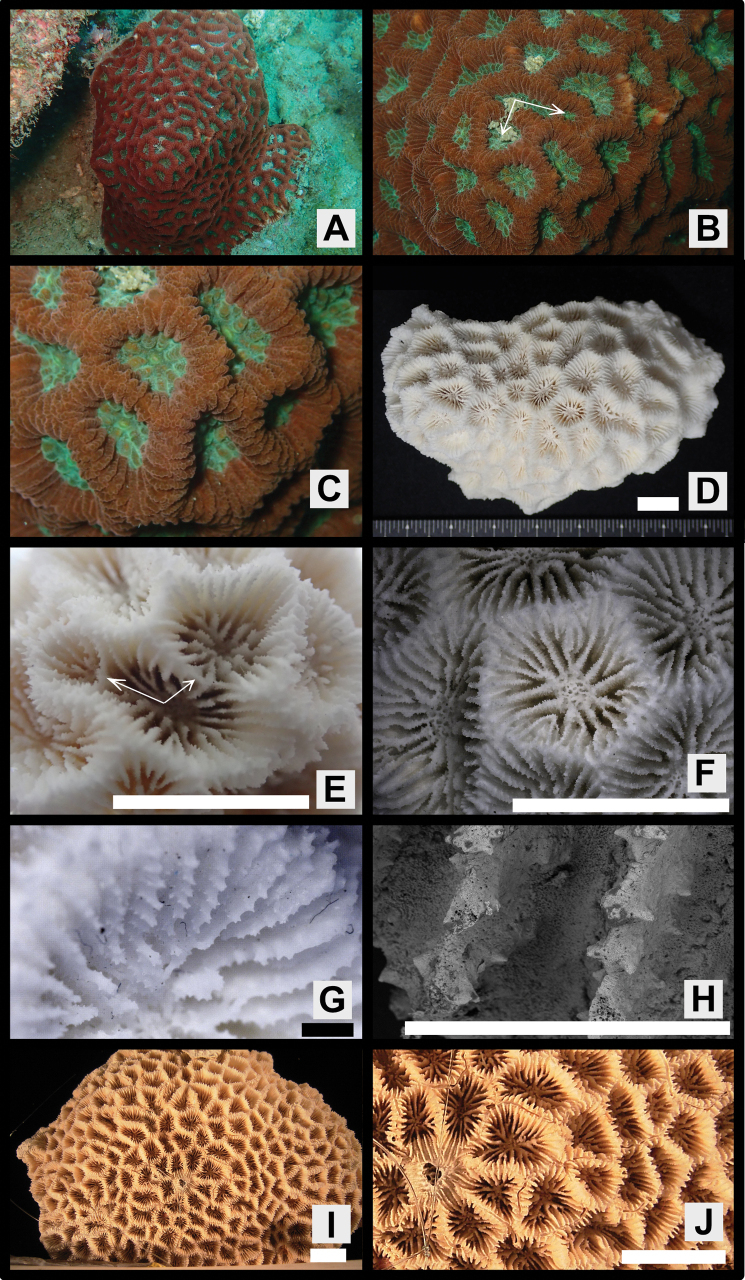
*Paragoniastreavariabilis* sp. nov. (Holotype: MUFS C588) and *Paragoniastreadeformis* (Holotype: MTQ G32487) **A–H***P.variabilis***A** colony *in situ***B** close up of corallite division in two directions **C** close up the colony *in situ***D** full scale of the skeletal specimen **E** corallite division in two directions from the skeletal specimen **F** corallites of the skeletal specimen **G** close up of one corallite of the skeletal specimen **H** septal teeth of the skeletal specimen **I–J***P.deformis***I** full scale of the skeletal specimen, **J** corallites of the skeletal specimen. Scale bar: 10 mm (**D, E, F, I, J**); 1 mm (**G, H**).

**Figure 3.**
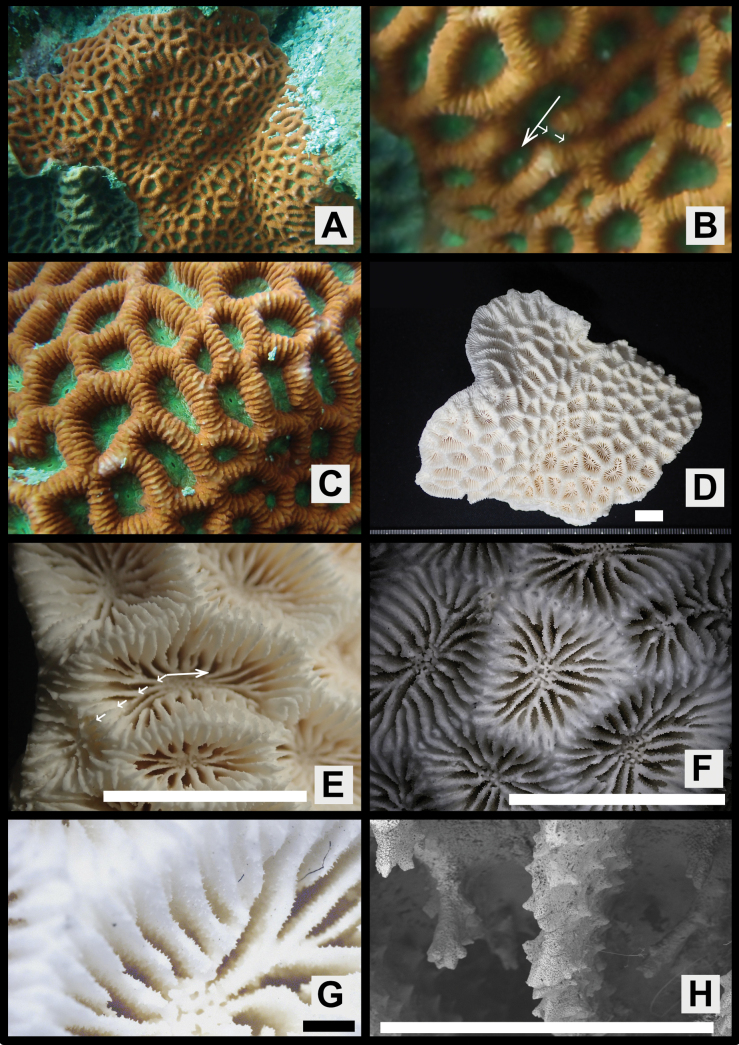
*Paragoniastreavariabilis* sp. nov. (Paratype: MUFS C78). **A** colony *in situ***B** close up of corallite division in two directions **C** close up the colony *in situ***D** full scale of the skeletal specimen **E** corallite division in two directions from the skeletal specimen **F** corallites of the skeletal specimen **G** close up of one corallite of the skeletal specimen **H** septal teeth of the skeletal specimen. Scale bar: 10 mm (**D, E**, **F**); 1 mm (**G**, **H**).

**Figure 4.**
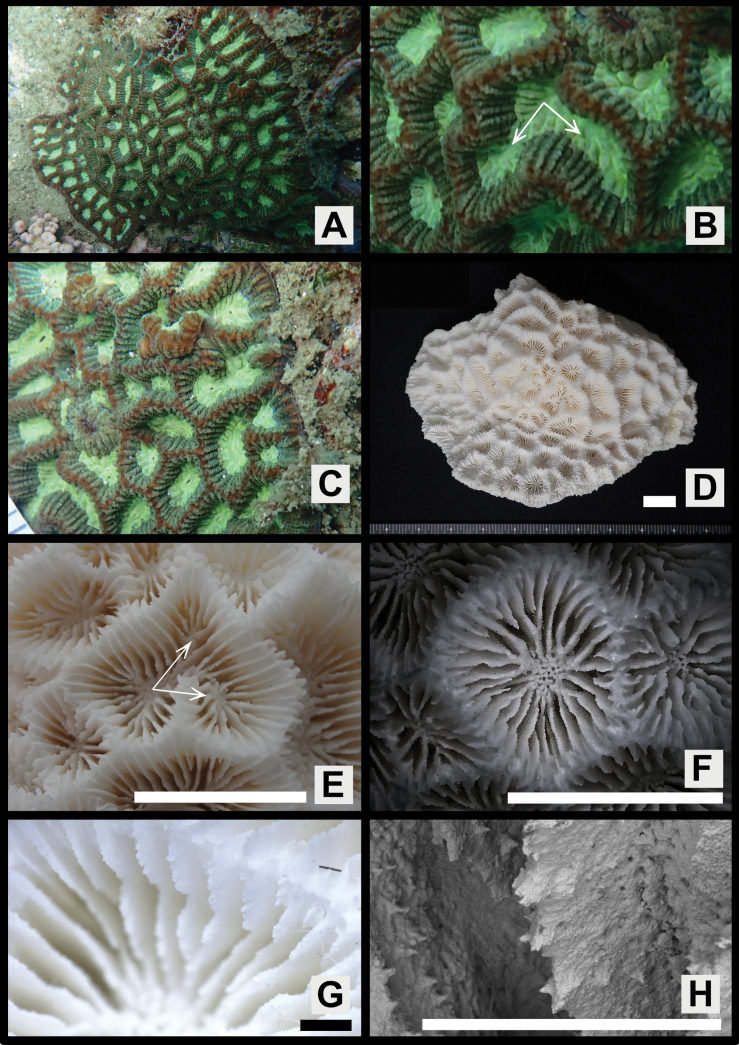
*Paragoniastreavariabilis* sp. nov. (Paratype: MUFS C585) **A** colony *in situ***B** close up of corallite division in two directions **C** close up the colony *in situ***D** full scale of the skeletal specimen **E** corallite division in two directions from the skeletal specimen **F** corallites of the skeletal specimen **G** close up of one corallite of the skeletal specimen **H** septal teeth of the skeletal specimen. Scale bar: 10 mm (**D, E, F**); 1 mm (**G, H**).

**Figure 5.**
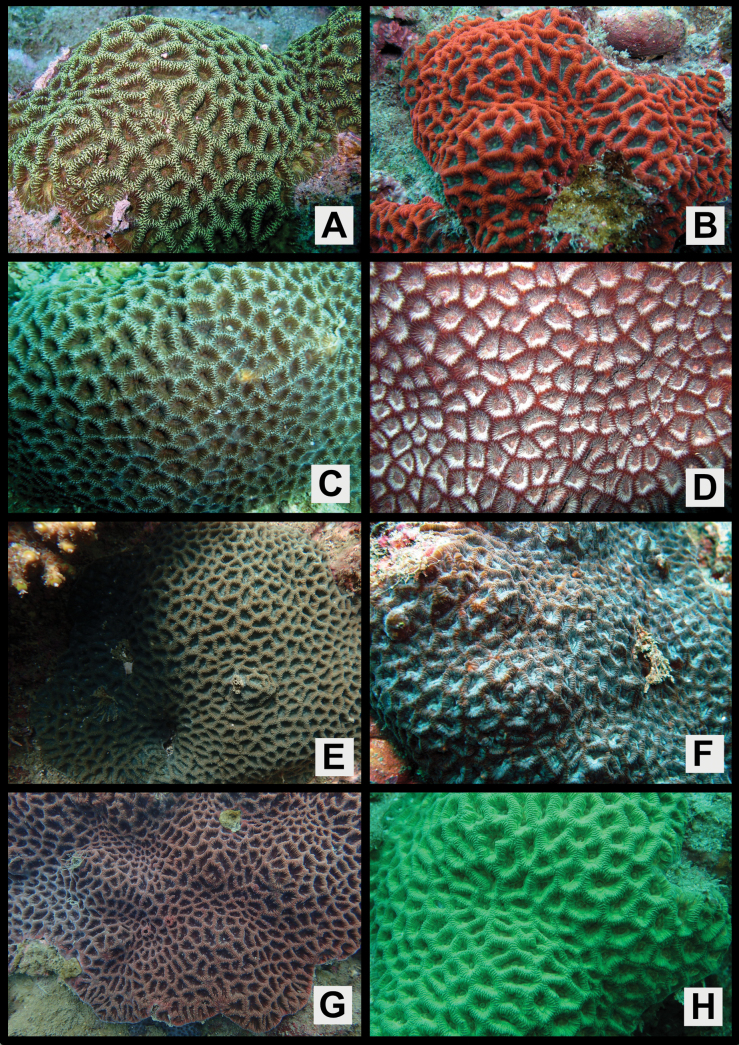
Color variation in life of *Paragoniastreavariabilis* sp. nov. **A**SMP-HC 894, Kushimoto, Wakayama, Japan **B**SMP-HC 1205, Kushimoto, Wakayama, Japan **C**SMP-HC 1749, Amakusa, Kumamoto, Japan **D**SMP-HC 1038, Kushimoto, Wakayama, Japan **E**MUFS C585, Amakusa, Kumamoto, Japan **F**SMP-HC 1208, Kushimoto, Wakayama, Japan **G** ASIZC0001666, Yehliu, New Taipei, Taiwan **H**SMP-HC 1203, Kushimoto, Wakayama, Japan.

Figs 2 , 3 , 4 , 5 Japanese name: henge-kamenoko-kikumeishi

Goniastreadeformis : Nishihira 1991: 254, 1 unnumbered fig.; Uchida and Soyama 1994: 65, figs 13, 14; Nishihira and Veron 1995: 345 (part), 3 unnumbered color figs; Veron 2000: 167 (part), 6; Nomura and Mezaki 2005: 35, pl. 1, fig. 28; Veron et al. 2016: part, unnumbered figs 4^th^, 6^th^ in the 1^st^ row, 3^rd^, 5^th^ in 2^nd^ row.
Paragoniastrea sp.: Sugihara et al. 2015: 153, 4 unnumbered figs; Huang et al. 2014: fig. 6I.
Paragoniastrea sp. HENGE *sensu*Nomura et al. 2016: 11; Nomura 2016: 44, figs A–F, 45 (part), figs A, D, E (B, C, F = P.australensis); Nomura et al. 2020: 40, fig. 20, G–I.

#### Type material.

***Holotype***: MUFS C588 (size 74 × 49 mm), sample MO417, Oshima Island, Nichinan, Miyazaki, Japan (31.527593°N, 131.401469°E), depth 9 m, 14 November 2021, coll. H. Fukami. ***Paratypes***: MUFS C78 (size 102 × 88 mm), sample number JP065, Sabiura, Kushimoto, Wakayama, Japan (33.464375°N, 135.785721°E), depth 5 m, 22 October 2012, coll. H. Fukami; MUFS C586 (size 90 × 66 mm), sample AM19-24, Satsuki, Amakusa, Kumamoto, Japan (32.457516°N, 130.207903°E), depth 4 m, 25 July 2019, coll. H. Fukami.

#### Other material.

**Japan.**SMP-HC 894, Takatomi Bay, Kushimoto, Wakayama, depth 10 m, 12 May 2003, coll. K. Nomura; SMP-HC 1038, Tosaki, Kushimoto, depth 6 m, 14 Dec 2003, coll. K. Nomura; SMP-HC 1203, 1205, 1208, Sabiura, Kushimoto, depth 3 m, 23 June 2005, coll. K. Nomura; SMP-HC 1749, Tanami, Kushimoto, depth 3 m, 24 Apr 2009, coll. K. Nomura; SMP-HC 1799, Takatomi Bay, Kushimoto, depth 6 m, 14 July 2009, coll. K. Nomura; SMP-HC 2957, Sabiura, Kushimoto, Wakayama, depth 9 m, 13 Dec 2015, coll. K. Nomura; SMP-HC 3049, Sabiura, Kushimoto, Wakayama, depth 10 m, 4 Feb 2016, coll. K. Nomura; MUFS C585, sample AM19-14, Satsuki, Amakusa, Kumamoto, depth 4 m, 25 July 2019, coll. H. Fukami; MUFS C587, sample NB92, Shimanourashima Island, Miyazaki, depth 13 m, 15 May 2015, coll. H. Fukami; MUFS C589, sample TK89, Takashima Island, Nagasaki, depth 3 m, 24 September 2021, coll. H. Fukami. **Taiwan.** ASIZC0001666, sample TWN45, Yehliu, New Taipei, depth 11 m, 4 July 2019, coll. H. Fukami (Suppl. material 1).

#### Comparative specimens.

*Paragoniastreaaustralensis*. MUFS C571, sample AM19-3, Amakusa, Kumamoto, Japan; MUFS C572, sample AM19-38, Amakusa, Kumamoto, Japan; MUFS C573, sample MO451, Oshima Island, Miyazaki, Japan; MUFS C574, sample MO457, Oshima Island, Miyazaki, Japan; MUFS C575, sample MO461, Oshima Island, Miyazaki, Japan; MUFS C576, sample NB96, Shimanourashima Island, Miyazaki, Japan; MUFS C577, sample NB148, Shimanourashima Island, Miyazaki, Japan; MUFS C578, sample OI10, Fukashima Island, Oita, Japan.

*Paragoniastreadeformis*. Holotype (MTQ G32487), Kushimoto, Wakayama, Japan; MUFS C579, sample AM19-18, Amakusa, Kumamoto, Japan; MUFS C580, sample AM19-21, Amakusa, Kumamoto, Japan; MUFS C581, sample AM19-26, Amakusa, Kumamoto, Japan; MUFS C582, sample AM19-36, Amakusa, Kumamoto, Japan; MUFS C583, sample MO450, Oshima Island, Miyazaki, Japan; MUFS C584, sample MO452, Oshima Island, Miyazaki, Japan; ASIZC0001667, sample TWN46, Yehliu, New Taipei, Taiwan; ASIZC0001669, sample TWN48, Yehliu, New Taipei, Taiwan.

Paragoniastreaaff.deformis. MUFS C590, sample AM19-19, Amakusa, Kumamoto, Japan; SMBL Cni-10321, sample JPO30, Shirahama, Wakayama, Japan; ASIZC0001691, sample TWN79, Yehliu, New Taipei, Taiwan.

#### Description.

Colonies massive or encrusting, surface smooth or rather uneven (Figs 2A, 3A, 4A, 5A–H).

Corallites intratentacular budding, arrangement mainly cerioid, rarely meandroid, 1–3 corallites lined up in one valley between corallite walls, percentage of having two corallites 2–19 (average ± SD: 8.2 ± 6.0) % and three corallites 0–8 (3.3 ± 3.3) %. Outline distorted quadrangular or pentagonal, usually compressed, calices 6.1–11.8 (average ± SD: 9.0 ± 1.6) mm in long diameter, moderately deep, height of calice relief (vertical distance from upper margin of corallite walls to columella) 2.0–4.3 (average 3.0 ± 0.7) mm (Figs 2B–F, 3B–F, 4B–F, Table 1).

**Table 1.** Morphological characteristics of *Paragoniastrea* spp. Left half is average (standard deviation), right half is value range (minimum to maximum). The measurements for each of the characteristics were taken from five corallites per specimen. For characteristics marked with an asterisk, three characters were measured from one corallite and the average of the three characters was treated as data from one corallite. Measurement in mm.

Characteristics	*P.variabilis* sp. nov.	*P.deformis*	P.aff.deformis	
	n=16	n=8	n=2 (AM19-19, JP030)	n=1 (TWN79)
Groove-and-tube structure	absent	present	absent	absent
Long diameter of calice	9.0 (1.6), 6.1–11.8	5.4 (0.8), 4.5–7.7	8.6 (1.0), 7.2–10.2	8.2 (0.4), 7.6–8.9
Long diameter of columella	1.6 (0.5), 0.9–2.8	1.5 (0.4), 1.24–2.3	1.9 (0.3), 1.4–2.4	1.8 (0.3), 1.3–2.1
height of calice relief	3.0 (0.7), 2.0–4.3	2.2 (0.6), 1.2–3.6	3.1 (0.7), 2.2–4.6	no data
Width of primary septa*	0.21 (0.02), 0.19–0.26	0.30 (0.02), 0.28–0.34	0.24 (0.02), 0.20–0.30	0.23 (0.02), 0.19–0.26
No. of septa per calice	44.7 (7.8), 28–62	33.1 (3.7), 29–44	66.5 (5.2), 59–76	36.6 (4.1), 32–44
No. of septa reaching collemulla	13.5 (2.4), 9–21	10.8 (1.7), 6–12	21.1 (1.4), 19–24	18.0 (2.1), 16–21
Length of septa*	3.7 (0.7), 2.7–5.1	3.5 (0.8), 1.4–4.1	3.8 (0.7), 2.8–5.1	3.2 (0.7), 2.1–4.4
No. of teeth on septa*	5.6 (0.9), 4–9	5.5 (0.6), 4–8	6.5 (0.5), 5–8	6.9 (0.5), 6–8
Distance between septal teeth*	0.31 (0.08), 0.1–0.6	0.34 (0.09), 0.2–0.6	0.29 (0.06), 0.2–0.4	0.20 (0.03), 0.1–0.3
Hight of paliform lobe*	1.18 (0.15), 0.68–1.37	1.14 (0.17), 0.87–1.37	1.12 (0.13), 0.86–1.27	no data
No. of teeth on paliform lobe*	2.1 (0.8), 1–4	2.3 (0.5), 1–4	3.0 (0.6), 2–4	1.4 (0.3), 1–2
No. of corallites in one valley	up to three, corallites	up to two, corallites	up to two, corallites	up to two, corallites
Percentage of two corallites in a valley	8.2 (6.0), 2–19	4.4 (2.3), 3–8	6.5 (0.8), 6–7	7
Percentage of three corallites in a valley	3.3 (3.3), 0–8	0	0	0

Septa formed by usually straight plates, steeply sloped along the corallite walls, numerous, present up to 4^th^ cycles, range of number 28–62 (average ± SD: 44.7 ± 7.8), length uneven, 2.7–5.1 (3.7 ± 0.7) mm length, width of primary septa thin, 0.19–0.26 (0.21 ± 0.02) mm wide, secondary and tertiary septa almost same width as primaries, quaternary septa usually faint. Primary and secondary septa connected to each other, and sometimes tertiary septa connected as well. Primary and secondary, and sometimes part of tertiary septa reaching columella (total number of septa reaching columella 9–21, average ± SD: 13.5 ± 2.4). Dorsal margin of septa with 4–9 (average ± SD: 5.6 ± 0.9) teeth. Teeth arranged vertically in single row, and surface covered with multiple spiny-like granules, sometimes with one short ridge on center of lateral faces. Distance between septal teeth is 0.1–0.6 (average ± SD: 0.31 ± 0.08) mm. Lateral faces of septa with sparsely distributed granules same as septal teeth (Figs 2E–G, 3E–G, 4E–G, Table 1).

Primary and secondary, and partial or all tertiary septa with rather distinct paliform lobes. Paliform lobes 0.68–1.37 (average ± SD: 1.18 ± 0.15) mm long vertically, uneven in shape and size, with 1–4 (2.1 ± 0.8) teeth on dorsal margin (Figs 2E–G, 3E–G, 4E–G, Table 1).

Columella rather large, spongy, formed by many entangled trabeculae, 0.9–2.8 (average ± SD: 1.6 ± 0.5) mm in long diameter (Figs 2F, 3F, 4F, Table 1).

Corallite walls joined between adjacent corallites, rather steeply sloped but standing upright even near the center of corallum, rather thin, groove-and-tube structure absent on walls joint (Figs 2D–F, 3D–F, 4D–F).

#### Color in life.

Colors are highly variable, usually soft bodies of corallite walls and calices are different. Corallite walls brown, greenish-brown, light greenish-brown, red or reddish-brown; calices brown, light brown, green, greenish-brown or light green (Figs 2A–C, 3A–C, 4A–C, 5A–H).

#### Distribution and habitat.

*Paragoniastreavariabilis* sp. nov. is known from warm-temperate, non-coral reef region in Japan (Wakayama, Kochi, Oita, Nagasaki, Kumamoto, Miyazaki and Kagoshima) and northern Taiwan (Fig. 1), found in shallow water at depths of 3–15 m, and is sympatric with all other species of the *Paragoniastrea* from Japan, namely *P.australensis*, *P.deformis* and P.aff.deformis.

#### Etymology.

The species is named from Latin *variabilis* (variable), in reference to the considerable color variations.

##### ﻿Remarks

###### ﻿Differences between species of *Paragoniastrea* based on morphological analysis

*Paragoniastrea* previously included three species, namely, *P.australensis*, *P.deformis* and *P.russelli* (Huang et al. 2014; Hoeksema and Cairns 2024). This study adds the new species *P.variabilis* sp. nov. (see Key to the species of *Paragoniastrea* below), which is separated from its congeners by following a combination of several characters: (1) The arrangement of corallites is dominantly cerioid, rarely meandroid; (2) Primary septa are not clearly thicker than secondary septa; and (3) the groove-and-tube structure is absent on corallite wall joint.

**Figure 6.**
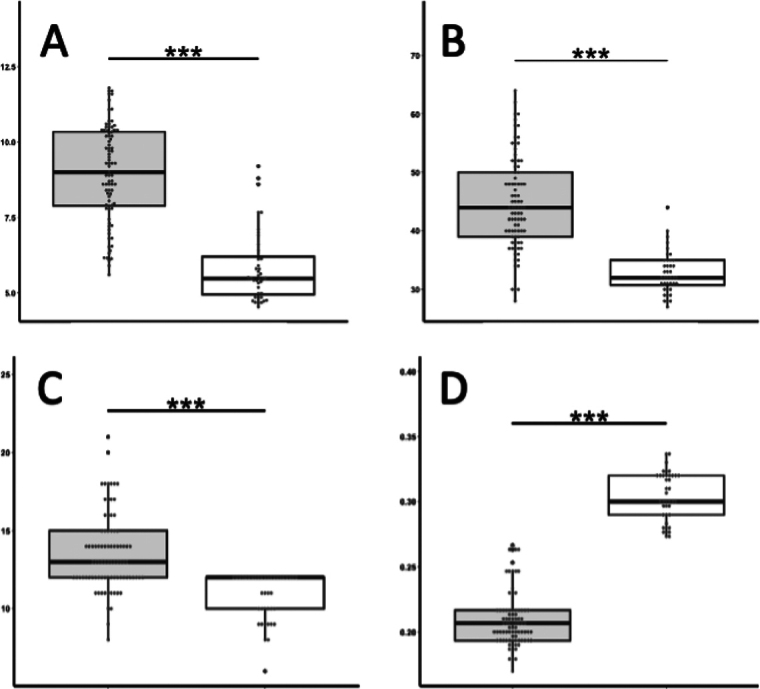
Morphological comparison between *P.variabilis* sp. nov. (left) and *P.deformis* (right) **A** long diameter of calice **B** number of septa per calice **C** number of septal reaching to columella **D** width of primary septa. Vertical bars indicate standard deviations, and the black horizontal line in the box-and-whisker chart indicates the median. ***: *p*<0.001.

**Figure 7.**
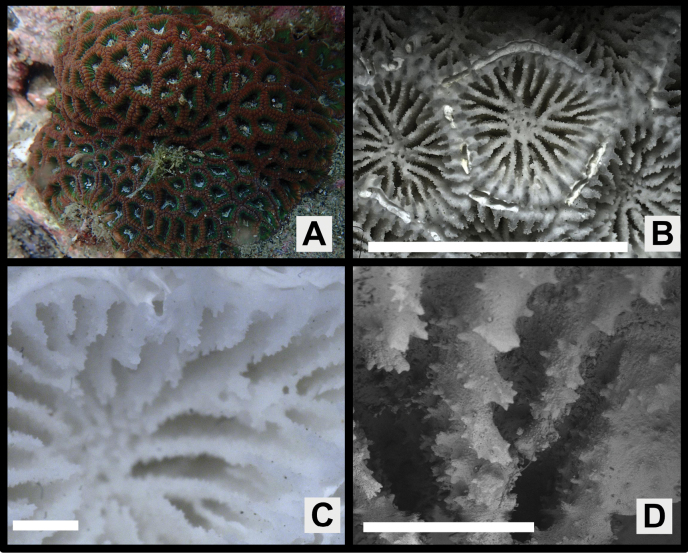
*Paragoniastreadeformis* (MUFS C580) **A** colony *in situ***B** corallites of the skeletal specimen **C** close up of one corallite of the skeletal specimen **D** septa and septal teeth. Scale bars: 10 mm (**B**); 1 mm (**C, D**).

The new species most closely resembles *P.deformis* in *Paragoniastrea* (Figs 2, 7). Both species have significant differences not only in the presence or absence of groove-and-tube structure but also in the following characteristics. *Paragoniastreavariabilis* sp. nov. has a larger average calice diameter (*p* < 0.001), average number of septa per calice (*p* < 0.001), and average number of septa reaching columella (*p* < 0.001) than *P.deformis*. In addition, *P.deformis* has wider primary septa than *P.variabilis* sp. nov. (*p* < 0.001) and up to two corallites in one valley between corallite walls, whereas *P.variabilis* sp. nov. has up to three corallites (Figs 2, 6, 7, Table 1).

Identifying the species, two specimens of sample AM19-19 (MUFS C590) from Kumamoto and JP030 (SMBL Cni-10321) from Wakayama, which were identified as *P.deformis* by Huang et al. (2014), had more septa per calice and more septa reaching columella than *P.deformis* and *P.variabilis* sp. nov. Although these specimens are morphologically different from known species, sufficient molecular phylogenetic evidence has not been obtained. Therefore, these specimens were tentatively treated as Paragoniastreaaff.deformis. In addition, one specimen, TWN79 (ASIZC0001691) from Taiwan, has similar morphological characteristics to *P.variabilis* sp. nov., but phylogenetic analysis showed this sample was included in the *P.deformis* clade in the IGR tree but formed an independent clade with specimen JP030 identified as P.aff.deformis, apart from *P.deformis* and *P.variabilis* sp. nov. in the ITS tree (see below molecular phylogenetic analysis). Thus, this specimen was treated as P.aff.deformis.

**Figure 8.**
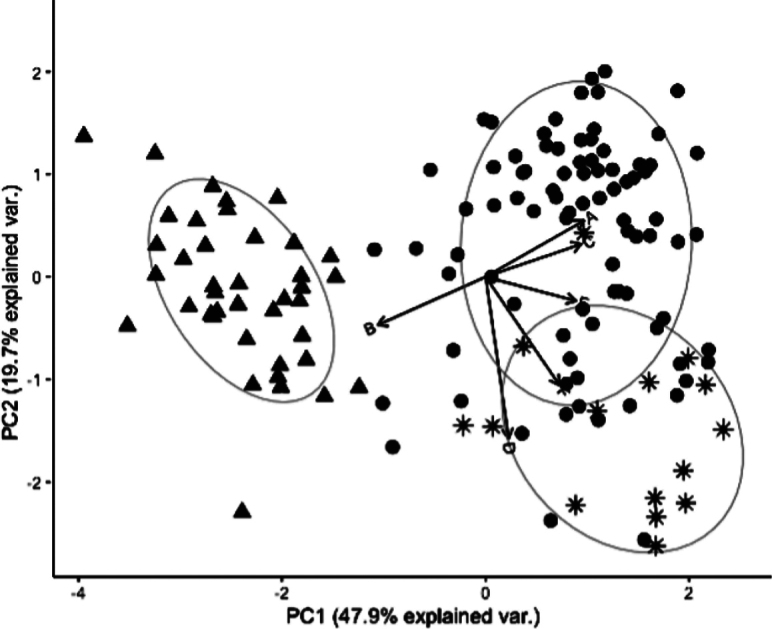
Principal component analysis on morphological characteristics of specimens in *P.variabilis* sp. nov. (black circle), *P.deformis* (black triangle) and P.aff.deformis (black asterisk). Arrows indicate long diameter of calice (**A**), width of primary septa (**B**), number of teeth on septa (**C**), long diameter of columella (**D**), number of septa per calice (**E**), number of septa reaching columella (**F**).

Principal component analysis using long diameter of columella, width of primary septa, number of teeth on septa, long diameter of columella, number of septa per calice, and number of septa reaching columella divided our samples into three groups. Among them, *P.variabilis* sp. nov. and *P.deformis* were separated along the PC1 axis, with PC1 explaining 47.9% of the morphological multivariate variance. Paragoniastreaaff.deformis was also distinct from *P.deformis* along the PC1 axis, but it partially overlapped with *P.variabilis* sp. nov. (Fig. 8).

###### ﻿Key to the species of *Paragoniastrea*

**Table:** 

1	Corallites arrangement procoid, cerioid or meandroid, width of primary septa clearly thicker than secondary septa	***P.russelli***
–	Corallites arrangement cerioid or meandroid, width of primary septa not clearly thicker than secondary septa	**2**
2	Corallites arrangement of meandroid dominant	***P.australensis***
–	Corallites arrangement cerioid dominant, meandroid rare	**3**
3	Groove-and-tube structure present on joint of corallite walls	***P.deformis***
–	Groove-and-tube structure absent on joint of corallite walls	***P.variabilis* sp. nov.**

###### ﻿Differences between species of *Paragoniastrea* based on molecular phylogenetic analysis

**Figure 9.**
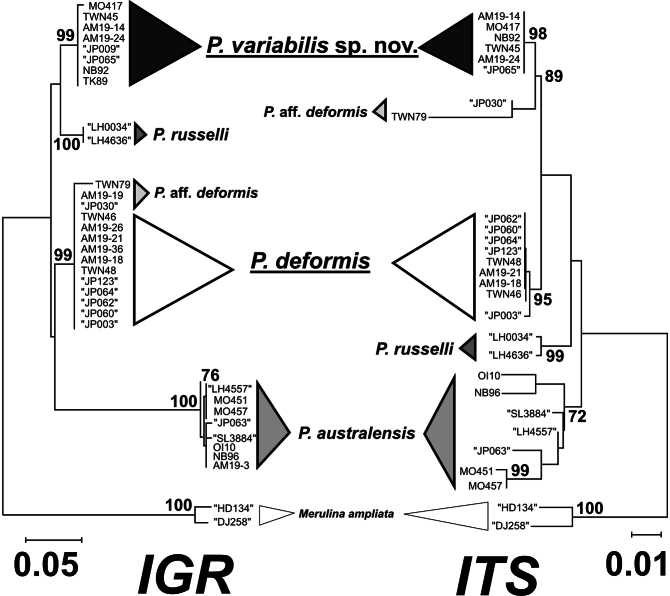
Molecular phylogenetic tree based on IGR (left) and ITS (right) for *Paragoniastrea* species. DNA sequences obtained from DDBJ were shown with double quotation marks with the specimen number.

For the IGR marker, the overall sequence length was 894 nucleotides with 102 polymorphic sites. The molecular phylogenetic tree of IGR showed that *P.variabilis* sp. nov. formed an independent clade from the other clades, including *P.deformis*, *P.australensis* and *P.russelli*. All three samples, AM19-19 (MUFS C590), TWN79 (ASTZC0001691), and JP030 (SMBL Cni-10321) of P.aff.deformis were included within the clade of *P.deformis* (Fig. 9).

For the ITS marker, the overall sequence length was 774 nucleotides with 69 polymorphic sites. The ITS tree showed that *P.deformis* and *P.variabilis* sp. nov. were also clearly separated into different clades (Fig. 9). Two samples (TWN79 and JP030) of P.aff.deformis formed an independent clade. The third sample (AM19-19) of P.aff.deformis could not be used for this analysis because of PCR amplification failure.

## ﻿Conclusion

*Paragoniastreavariabilis* sp. nov. had been considered an intraspecific morphological variant of *P.deformis* because of their sympatric distributions and similar morphology (see synonymy). In this study, the results of morphological and molecular phylogenetic analyses showed that *P.variabilis* sp. nov. and *P.deformis* are clearly distinct from each other. Thus, we describe *P.variabilis* sp. nov. as a new species of *Paragoniastrea*.

**Figure 10.**
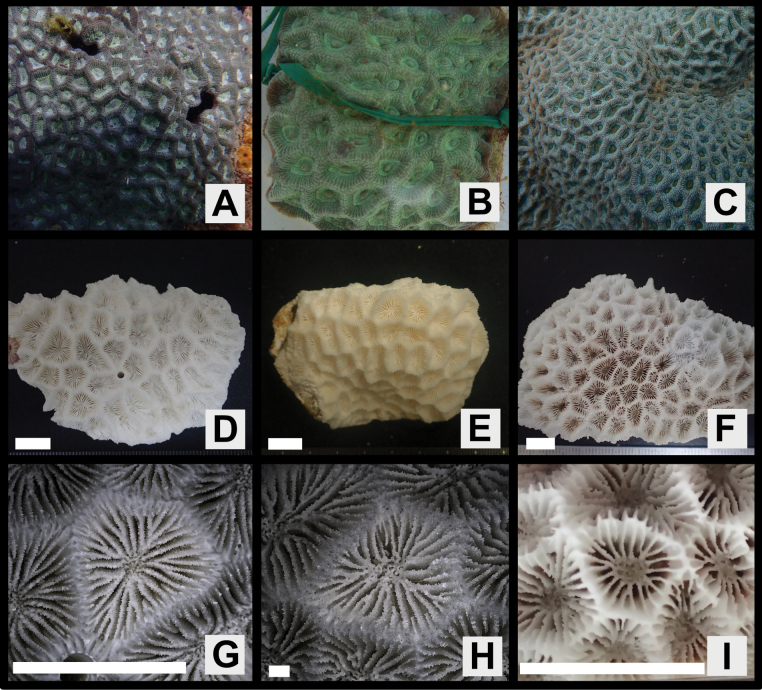
Paragoniastreaaff.deformis**A–C** colonies *in situ***D–F** full scale of the skeletal specimens **G–I** corallites of the skeletal specimens **A, D, G**MUFS C590 (AM19-19), Amakusa, Kumamoto Japan **B, E, H** ASIZC0001691 (TWN79), Yehliu, New Taipei, Taiwan **C, F, I**SMBL Cni-10321 (JP030), Kushimoto, Wakayama, Japan. Scale bar: 10 mm (**D–G, I**); 1 mm (**H**).

Finally, the taxonomic position of P.aff.deformis (Fig. 10) remains unclear because of insufficient morphological and phylogenetic studies, so better sampling and analyses will be needed to clarify its species status.

## Supplementary Material

XML Treatment for Paragoniastrea variabilis

## ﻿Additional information

### Author ORCIDs

Hironobu Fukami https://orcid.org/0000-0002-7647-6668

### Data availability

All of the data that support the findings of this study are available in the main text or Supplementary Information.

## Supplementary materials

Supplementary material 1 List of specimens that we collected or used in this study Data type xlsx Explanation note Sample information including sampling sites and DNA sequences. https://binary.pensoft.net/file/1078430This dataset is made available under the Open Database License (http://opendatacommons.org/licenses/odbl/1.0/). The Open Database License (ODbL) is a license agreement intended to allow users to freely share, modify, and use this Dataset while maintaining this same freedom for others, provided that the original source and author(s) are credited.Daigo Kishi, Keiichi Nomura, Yoko Nozawa, Seiji Arakaki, Hironobu Fukami

Supplementary material 2 List of species of *Paragoniastrea* referred from Huang et al. (2014) and used in molecular phylogenetic analysis Data type xlsx Explanation note Sample list of referred DNA sequences. https://binary.pensoft.net/file/1078431This dataset is made available under the Open Database License (http://opendatacommons.org/licenses/odbl/1.0/). The Open Database License (ODbL) is a license agreement intended to allow users to freely share, modify, and use this Dataset while maintaining this same freedom for others, provided that the original source and author(s) are credited.Daigo Kishi, Keiichi Nomura, Yoko Nozawa, Seiji Arakaki, Hironobu Fukami

## References

[B1] Fukami H Budd AF Levitan DR Jara J Kersanach R Knowlton N (2004) Geographic differences in species boundaries among members of the *Montastraeaannularis* complex based on molecular and morphological markers. Evolution 58: 324–337. 10.1111/j.0014-3820.2004.tb01648.x 15068349

[B2] Hoeksema BW Cairns S (2024) World List of Scleractinia. *Paragoniastrea* Huang, Benzoni and Budd, 2014. [World Register of Marine Species] https://www.marinespecies.org/aphia.php?p=taxdetails&id=817176 [on 2024-01-25]

[B3] Huang D Benzoni F Arrigoni R Baird AH Berumen ML Bouwmeester J Chou LM Fukami H Licuanan WY Lovell ER Meier R Todd PA Budd AF (2014) Towards a Phylogenetic classification of reef corals: The Indo-Pacific genera *Merulina*, *Goniastrea* and *Scapophyllia* (Scleractinia, Merulinidae). Zoologica Scripta 43(5): 531–548. 10.1111/zsc.12061

[B4] Katoh K Rozewicki J Yamada DY (2019) MAFFT online service: Multiple sequence alignment, interactive sequence choice and visualization. Briefings in Bioinformatics 20(4): 1160–1166. 10.1093/bib/bbx108 28968734 PMC6781576

[B5] Kuraku S Zmasek CM Nishimura O Katoh K (2013) aLeaves facilitates on-demand exploration of metazoan gene family trees on MAFFT sequence alignment server with enhanced interactivity. Nucleic Acids Research 41(W1): W22–W28. 10.1093/nar/gkt389 PMC3692103 23677614

[B6] Mitsuki Y Isomura N Nozawa Y Tachikawa H Huang D Fukami H (2021) Distinct species hidden in the widely distributed coral *Coelastreaaspera* (Cnidaria, Anthozoa, Scleractinia). Invertebrate Systematics 35(8): 876–891. 10.1071/IS21025

[B7] Nishihira M (1991) Field guide to hermatypic corals of Jama. First enlarged edition. Tokai University Press, Tokyo, 264 pp. [In Japanese]

[B8] Nishihira M Veron JEN (1995) Hermatypic corals of Japan. Kaiyusya, Tokyo, 439 pp. [In Japanese]

[B9] Nomura K (2016) Zooxanthellate scleractinian corals of Kushimoto II, Vacatina. Marine Pavilion, supplement 6, 69 pp. [In Japanese] http://www.kushimoto.co.jp/marinepavilion/data/sup6.pdf

[B10] Nomura K Mezaki T (2005) Reef building corals from Ostuki, Kochi Prefecture, Japan. Kuroshio Biosphere 2: 29–41. [In Japanese] https://kuroshio.or.jp/wp-content/uploads/2019/06/KuroshioBiosphere_02_29-41_NomuraMezaki.pdf

[B11] Nomura K Fukami H Zayasu Y Shimada G Kitano Y Yokochi H Shimoike K Tachikawa H Oku Y Suzuki G Kajiwara K (2016) Revision of Zooxanthellate scleractinian corals of Kushimoto. Marine Pavilion, supplement 4, 20 pp. [In Japanese] http://www.kushimoto.co.jp/marinepavilion/data/sup4.pdf

[B12] Nomura K Yokochi H Kimura T Kajiwara K Nojima S Arakaki S (2020) Zooxanthellate scleractinian corals collected from the Amakusa Islands, Kumamoto, western Kyushu, Japan. Coastal Ecosystems 7: 1–52.

[B13] Quek ZBR Jain SS Richards ZT Arrigoni R Benzoni F Hoeksema BW Carvajal JI Wilson NG Baird AH Kitahara MV Seiblitz IGL Vaga CF Huang D (2023) A hybrid-capture approach to reconstruct the phylogeny of Scleractinia (Cnidaria: Hexacorallia). Molecular Phylogenetics and Evolution 186: 107867. 10.1016/j.ympev.2023.107867 37348770

[B14] Randall RH Eldredge LG (1976) Skeletal modification by a polychaete annelid in some scleractinian corals. Coelenterate Ecology and Behavior. Springer, 453–465. 10.1007/978-1-4757-9724-4_48

[B15] Rosen BR (1968) An account of a pathologic structure in the Faviidae (Anthozoa): A revision of *Faviavalenciennesii* (Edwards & Haime) and its allies (Pis.1–8). Bulletin of the British Museum (Natural History). Zoology 16: 323–352. 10.5962/p.314176

[B16] Sugihara K Nomura K Yokochi H Shimoike K Kajiwara K Suzuki G Zayasu Y Dewa N Fukami H Kitano Y Matsumoto H Mezaki T Nagata S Tachikaw H Kimura T (2015) Zooxanthellate scleractinian corals of Tanegashima Island. Center for Environmental Biology and Ecosystem Studies, National Institute for Environmental Studies, Japan, 197 pp. [In Japanese]

[B17] Tamura K Stecher G Kumar S (2021) MEGA11: Molecular Evolutionary Genetics Analysis Version 11. Molecular Biology and Evolution 38(7): 3022–3027. 10.1093/molbev/msab120 33892491 PMC8233496

[B18] Uchida H Soyama I (1994) Rochy reef animals. In: Okutani T (Ed.) Yama-kei, Tokyo, 367 pp. [In Japanese]

[B19] Veron JEN (1990) New Scleractinia from Japan and other Indo-West Pacific countries. Galaxea 9: 95–173.

[B20] Veron JEN (2000) Corals of the World, Vol. 3. Australian Institute of Marine Science, Townsville, 490 pp.

[B21] Veron JEN Stafford-Smith MG Turak E DeVantier LM (2016) Corals of the World. http://www.coralsoftheworld.org/species_factsheets/species_factsheet_summary/goniastrea-deformis/ [Accessed 18 Feb 2024]

[B22] Wei NWV Wallace CC Dai CF Pillay KM Chen CA (2006) Analyses of the ribosomal internal transcribed Spacers (ITS) and 5.8 S gene indicate that extremely high rDNA heterogeneity is a unique feature in the scleractinian coral genus *Acropora* (Scleractinia; Acroporidae). Zoological Studies 45: 404–418. http://zoolstud.sinica.edu.tw/Journals/45.3/404.pdf

[B23] Wilfredo YL Emmi BC (2003) Range extensions of Japanese Scleractinia to the Philippines. Galaxea. Journal of the Coral Reef Studies 5: 63–67. 10.3755/jcrs.2003.63

